# Sociodemographic inequalities in product-specific tobacco use in Myanmar: a nationally representative study of the 2015–2016 demographic and health survey

**DOI:** 10.3389/fpubh.2026.1839321

**Published:** 2026-06-23

**Authors:** Soe Sandi Tint, Kanittha Thaikla, Aung Tun, Kong Sam An, Jiraporn Laoung-on, Hlaing Thaw Dar, Myo Zin Oo

**Affiliations:** 1 Global Health and Chronic Conditions Research Center, Chiang Mai University, Chiang Mai, Thailand; 2 Department of Family Medicine, Faculty of Medicine, Chiang Mai University, Chiang Mai, Thailand; 3 Research Institute for Health Sciences, Chiang Mai University, Chiang Mai, Thailand; 4 Tun Khit Foundation, Yangon, Myanmar; 5 Department of Mental Health and Substance Abuse, Ministry of Health, Phnom Penh, Cambodia; 6 Public Health and Community Medicine Department, IMU University School of Medicine, Kuala Lumpur, Malaysia

**Keywords:** demographic and health survey, dual tobacco use, Myanmar, smoked tobacco, smokeless tobacco, tobacco use

## Abstract

**Introduction:**

Tobacco use remains a major preventable cause of disease in low- and middle-income countries, and nationally representative evidence distinguishing tobacco product types in Myanmar remains limited. We aimed to provide nationally representative, product-specific estimates of smoked, smokeless, and dual tobacco use among individuals aged 15–49 years in Myanmar using data from the 2015–2016 Demographic and Health Survey, and to examine sociodemographic inequalities in tobacco use patterns.

**Methods:**

We conducted a cross-sectional secondary analysis using data from the 2015–2016 Myanmar Demographic and Health Survey (MDHS), the most recent nationally representative household survey with adult tobacco use indicators in Myanmar. The sample included 17,620 individuals aged 15–49 years with complete tobacco use data. Tobacco use was classified as no use, smoked tobacco only, smokeless tobacco only, or dual use. Weighted descriptive statistics and survey-weighted multivariable multinomial logistic regression adjusted for age, sex, education, residence, marital status, occupation, wealth quintile, and mass media exposure were used to assess associations with sociodemographic factors, reporting adjusted relative risk ratios (ARRRs) with 95% confidence intervals (CIs). Statistical significance was defined as *p*<0.05.

**Results:**

Overall, 86.3% reported no tobacco use; smoked tobacco use was 13.0%, whereas smokeless (0.4%) and dual use (0.3%) were rare. Older age (40–49 years) was associated with higher likelihood of smoked tobacco use (ARRR = 3.58, 95% CI 2.70, 4.74, *p* < 0.001). Men had a substantially higher likelihood of smoked tobacco use (ARRR = 21.96, 95% CI 18.38, 26.22, *p* < 0.001) and higher likelihood of smokeless and dual tobacco use, although estimates for the latter categories should be interpreted cautiously because of small numbers. Higher education and wealth were associated with lower likelihood of smoked tobacco use. Being not currently married or cohabiting was associated with higher likelihood of smoked tobacco use and dual use.

**Conclusions:**

Smoked tobacco predominated among adults aged 15–49 years, while smokeless (chewing tobacco and snuff) and dual use were rare. Marked sociodemographic inequalities highlight the product-specific, equity-oriented tobacco control and updated surveillance. Findings should be interpreted considering the cross-sectional design, potential self-report bias, limited generalizability due to restriction to individuals aged 15–49 years, and the 2015–2016 survey period.

## 1 Introduction

Tobacco use remains a major preventable public health problem and continues to pose considerable challenges for health systems in low- and middle-income countries (LMICs) (1). Globally, most people who use tobacco live in LMICs, which bear a disproportionate share of the tobacco-related disease burden (2). In Myanmar, strengthening the evidence base to inform tobacco control requires nationally representative surveillance data to monitor population-level patterns of tobacco use and to support evidence-based policy responses aimed at reducing population-level tobacco exposure. Such data are essential for identifying subgroups with elevated exposure, assessing inequalities in tobacco use, and characterizing patterns of use across sociodemographic groups. Understanding who uses tobacco, and in what forms, provides a critical foundation for developing targeted and equitable tobacco control strategies.

Importantly, tobacco use is a heterogeneous behavior encompassing multiple product types with distinct patterns of exposure, risk profiles, and regulatory implications (3). Smoked and smokeless tobacco products differ in their modes of use and policy relevance (4, 5), while dual tobacco users represent a subgroup with compounded exposure and potentially greater vulnerability (6). In settings such as Myanmar, where multiple tobacco products are used, analyzing tobacco use as a single behavior may overlook important differences in patterns of use across sociodemographic groups. Such aggregation may also lead to exposure misclassification and dilute observed associations with population characteristics. Product-specific classification provides a more granular analytical framework for examining heterogeneity in tobacco use across population subgroups.

Population-based surveillance systems play a central role in monitoring tobacco use and enabling comparable estimates across countries to support evidence-based tobacco control policy (7). In Myanmar, nationally representative data sources for adult tobacco use remain limited, with the 2015–2016 Demographic and Health Survey (DHS) providing the most recent comprehensive population-level data that include tobacco use indicators among adults (8), complemented by periodic youth-focused surveillance such as the Global Youth Tobacco Survey (9); however, more recent nationally representative data on adult tobacco use remain scarce. In this context, analyses based on the Myanmar Demographic and Health Survey (MDHS) 2015–2016 provide important baseline evidence on population-level patterns of tobacco use and their sociodemographic distribution in Myanmar. These data are valuable for informing tobacco control strategies and for establishing a reference point for future surveillance, although the findings should be interpreted within the survey period rather than as estimates of current national prevalence. Importantly, analyses of the MDHS can provide nationally representative baseline estimates of product-specific tobacco use in Myanmar while highlighting sociodemographic inequalities and limitations in current surveillance.

Within this context, previous analyses of the MDHS 2015–2016 have examined tobacco use in combination with betel quid chewing and reported sex-specific estimates (10). However, product-specific patterns of smoked, smokeless, and dual tobacco use remain underexplored using nationally representative data in Myanmar. A product-specific approach may help characterize variation in tobacco use patterns across population subgroups and provide baseline evidence on the distribution of different tobacco use patterns among individuals aged 15–49 years. Therefore, our study aimed to provide nationally representative, product-specific estimates of smoked, smokeless, and dual tobacco use among individuals aged 15–49 years in Myanmar using data from the 2015–2016 Demographic and Health Survey, and to examine sociodemographic inequalities in tobacco use patterns.

## 2 Methods

### 2.1 Study design and setting

We conducted a cross-sectional analytical design based on secondary analysis of nationally representative survey data from Myanmar. The analysis used data from the 2015–2016 Myanmar Demographic and Health Survey (MDHS) (8), the most recent nationally representative survey with adult tobacco use indicators in Myanmar, which was implemented nationwide across all states and regions and covered both urban and rural areas. Fieldwork for the survey was conducted between December 2015 and July 2016. The MDHS was implemented by the Ministry of Health and Sports with technical support from ICF under The DHS Program. Data collection was carried out through face-to-face interviews by trained field teams following standardized survey protocols and quality assurance procedures. The MDHS 2015–2016 represents the most recent nationally representative dataset with adult tobacco use indicators currently available for Myanmar.

### 2.2 Data source and sampling procedure

Data were drawn from the MDHS 2015–2016 (8), a nationally representative household survey designed to generate estimates at the national level as well as by state/region and urban-rural residence. The MDHS followed a two-stage, stratified cluster sampling framework based on the 2014 Myanmar Population and Housing Census cartographic sampling frame. Primary sampling units (enumeration areas) were first selected within strata defined by state/region and place of residence, resulting in 442 clusters across 30 strata (123 urban and 319 rural). Households were then chosen using systematic sampling methods from revised household listings within each selected cluster. On average, approximately 30 households were sampled per cluster, yielding a total of 13,260 households. Sampling relied on updated household listings, and selected households were not substituted. The women's survey was administered in all sampled households, whereas the men's survey was conducted in a subset of households, consistent with Demographic and Health Survey (DHS) field protocols. Individual sample weights provided in the DHS dataset were applied to account for differential selection probabilities and non-response. All analyses incorporated sampling weights and accounted for clustering and stratification to address unequal selection probabilities and the complex survey design.

### 2.3 Study population

**Figure 1:**
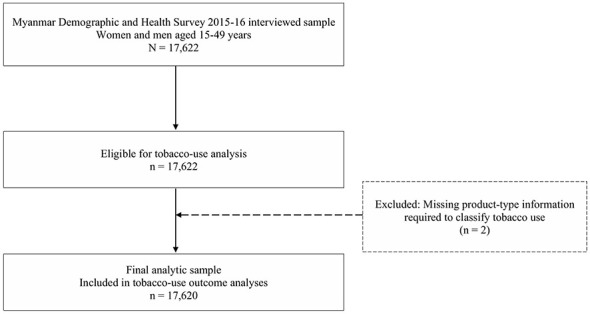
Study flow diagram. Among 17,622 women and men aged 15–49 years interviewed in the 2015–16 Myanmar Demographic and Health Survey, two respondents were excluded due to missing information required to classify tobacco-use status. The final analytic sample therefore comprised 17,620 respondents.

The study population included all women and men aged 15–49 years who completed interviews in the MDHS 2015–2016 (8). In the achieved sample, 12,885 women and 4,737 men were interviewed, yielding a total of 17,622 respondents. Of these, 17,620 respondents had complete information required to classify current tobacco-use status and were included in the present analysis; two respondents (< 0.1%) were excluded due to missing product-type information (Figure 1). Survey weights were applied in all analyses to ensure national representativeness. The analytic sample was restricted to respondents with complete data on tobacco use variables.

### 2.4 Outcome variable

The outcome variable in this study was current tobacco use pattern, derived from three questions in the MDHS 2015–2016 (8):

(a) “Do you currently smoke cigarettes?” (yes/no);

(b) “Do you currently smoke or use any other type of tobacco?” (yes/no); and

(c) “What (other) type of tobacco do you currently smoke or use?” with response options including pipe/cigar/cheroot, chewing tobacco, snuff, and other (specify).

The screening question (b) was not used for outcome classification because it does not distinguish between smoked and smokeless tobacco products. Therefore, classification of tobacco use patterns was based on reported product types (question c) and cigarette smoking (question a). We constructed two binary indicators reflecting any smoked tobacco use and any smokeless tobacco use. Smoked tobacco use was coded as 1 if respondents reported currently smoking cigarettes (question a) or using smoked products (pipe, cigar, or cheroot) (question c). Smokeless tobacco use was coded as 1 if respondents reported current use of chewing tobacco or snuff (question c). Although the MDHS includes a variable for betel quid use with tobacco, this variable contained no usable observations in the analyzed dataset and was not available for male respondents; therefore, it was excluded from the present analysis to ensure consistency across the study population. Responses coded as “other (specify)” in the product list (question c) were not classified as smoked or smokeless tobacco because the specific product type is not available in the DHS recode files; these responses were therefore excluded from product-type classification to minimize potential misclassification. The two indicators were combined to generate a four-category outcome: no tobacco use, smoked tobacco use only, smokeless tobacco use only, and dual tobacco use. Dual use was defined as concurrent use of at least one smoked and one smokeless tobacco product. This categorization follows established approaches in global tobacco surveillance and enables separate estimation of correlates of smoked, smokeless, and dual tobacco use while preserving heterogeneity in tobacco use patterns.

### 2.5 Explanatory variables

The explanatory variables in this study were selected based on their availability in the MDHS 2015–2016 (8) and prior evidence on sociodemographic correlates of tobacco use (11). These included age, sex, educational attainment, place of residence, marital status, occupation, household wealth quintile, and exposure to mass media. All variables were derived from standard variables provided in the DHS dataset and categorized for analysis.

### 2.6 Statistical analysis

All analyses were performed using Stata version 15. Descriptive statistics were used to summarize participants' background characteristics, and the distribution of tobacco use patterns, with results presented as unweighted frequencies and weighted percentages with 95% confidence intervals (CI). Bivariate associations between covariates and tobacco use patterns were assessed using design-adjusted χ^2^ tests.

Multinomial logistic regression was employed to estimate the associations between independent variables and tobacco use patterns, which were classified into four mutually exclusive categories: no tobacco use, smoked tobacco only, smokeless tobacco only, and dual use. In this study, sociodemographic inequalities refer to differences in the distribution of tobacco use patterns across population subgroups, as quantified using survey-weighted regression models. “No tobacco use” was specified as the reference category. Both unadjusted and adjusted models were fitted, and results are presented as relative risk ratios (RRRs) and adjusted relative risk ratios (ARRRs) with 95% CI. Unadjusted bivariable multinomial regression results are presented in Supplementary Table S1. All covariates were selected *a priori* based on theoretical relevance and prior evidence on sociodemographic correlates of tobacco use and were entered simultaneously into the multivariable model to control for potential confounding. Sex was included as a key covariate in the multinomial regression models to account for substantial gender differences in tobacco use in Myanmar while enabling estimation of overall population-level product-specific tobacco use patterns.

Multicollinearity was assessed using variance inflation factors (VIFs) derived from an equivalent regression model, given that VIFs are not directly available following survey-weighted multinomial regression. No evidence of problematic multicollinearity was observed (mean VIF = 2.09; all VIFs < 3.00). Overall model adequacy was evaluated using a likelihood ratio test comparing the fitted model with a null model (LR χ^2^ = 4,373.38, *p* < 0.001).

All analyses accounted for the complex survey design using appropriate survey commands, incorporating sampling weights, clustering, and stratification. This approach adjusts for unequal probabilities of selection and intra-cluster correlation, yielding nationally representative estimates. Unweighted cell counts were examined to assess the stability of estimates in small subgroups, and categories with fewer than 25 observations were interpreted with caution. Estimates affected by complete or near-complete separation were retained for transparency but were not substantively interpreted. This practice is commonly applied in large-scale tobacco surveillance studies, including those conducted under the Global Adult Tobacco Survey (GATS) and the Global Tobacco Surveillance System (GTSS). Household wealth quintiles were based on the DHS-constructed wealth index derived using principal component analysis of household assets and characteristics (8). Statistical significance was defined as *p* < 0.05.

### 2.7 Ethical approval

The MDHS 2015–2016 (8) was implemented in accordance with international ethical standards for research involving human participants, including independent ethical review and informed consent procedures, and is consistent with the ethical principles outlined in the Declaration of Helsinki. The survey protocol was reviewed and approved by the Ethics Review Committee on Medical Research involving Human Subjects, Department of Medical Research, Ministry of Health and Sports, Myanmar, as well as the Institutional Review Board of ICF. Written informed consent was obtained from all participants, and measures were in place to protect respondent confidentiality. Our study represents a secondary analysis of anonymized MDHS data. Authorization to access the dataset was granted by the DHS Program on 3 February 2026 following approval of the study proposal submitted through the DHS data access system. As the dataset is publicly available and de-identified, no additional institutional ethical approval was required for this secondary analysis.

## 3 Results

Table 1 presents the background characteristics of the respondents. Among the participants, 30.5% were aged 30–39 years, 73.1% were female, and 71.0% lived in rural areas. Regarding educational attainment, 39.7% had completed primary education, while 60.8% were married. The largest occupational group was unskilled manual workers (25.8%), and 21.8% of respondents belonged to the highest wealth quintile. More than two-thirds of participants (68.9%) reported high exposure to mass media.

**Table 1:** Background characteristics of the respondents (*N* = 17,620).

Variables	Unweighted number (*N*)	Weighted percentage (%)	Weighted 95% CI
Age			
15–19	2,603	14.4	13.8, 15.1
20–29	5,149	29.0	28.1, 29.8
30–39	5,233	30.5	29.6, 31.4
40–49	4,635	26.1	25.3, 27.1
Sex			
Female	12,883	73.1	72.5, 73.8
Male	4,737	26.9	26.2, 27.5
Education (*n* = 17,618)			
No education	2,151	12.4	11.0, 13.9
Primary	6,759	39.7	38.0, 41.4
Secondary	7,060	38.5	36.9, 40.2
Higher	1,648	9.4	8.5, 10.5
Residence			
Rural	12,514	71.0	69.6, 72.3
Urban	5,106	29.0	27.7, 30.4
Marital status			
Never married	5,841	33.6	32.6, 34.7
Married	10,784	60.8	59.7, 61.9
Not currently married or cohabiting^a^	995	5.6	5.2, 6.0
Occupation (*n* = 17,581)			
Not working	4,002	21.9	20.6, 23.2
Agriculture/self-employed	3,134	17.7	15.7, 20.0
Clerical/sales/services	3,035	17.6	16.3, 18.9
Professional/technical/managerial	1,181	5.9	5.0, 7.0
Skilled manual	1,848	11.1	9.9, 12.4
Unskilled manual	4,381	25.8	23.7, 27.9
Wealth quintile			
Lowest	3,268	17.9	16.3, 19.7
Second	3,384	18.9	17.5, 20.3
Middle	3,647	20.5	19.1, 22.0
Fourth	3,734	20.9	19.2, 22.7
Highest	3,587	21.8	19.7, 23.9
Exposure to mass media			
No exposure	2,123	11.8	10.5, 13.2
Low exposure	3,650	19.3	18.3, 20.4
High exposure	11,847	68.9	67.2, 70.5

Numbers (*N*) are unweighted counts, and percentages (%) are weighted to represent the national population with 95% confidence intervals (CI). This table presents descriptive statistics only; no inferential statistical tests were applied. ^a^Includes widowed, divorced, and separated/ no longer living together.

Table 2 presents the patterns of tobacco use among the respondents. Overall, 86.3% reported no current tobacco use, while 13.0% used smoked tobacco. Smokeless tobacco use (0.4%) and dual tobacco use (0.3%) were relatively uncommon in the study population.

**Table 2:** Patterns of smoked, smokeless, and dual tobacco use among respondents (*N* = 17,620).

Tobacco use	Unweighted number (*N*)	Weighted percentage (%)	Weighted 95% CI
No tobacco use	15,033	86.3	85.6, 87.0
Smoked tobacco use	2,472	13.0	12.3, 13.7
Smokeless tobacco use	64	0.4	0.2, 0.5
Dual tobacco use	51	0.3	0.2, 0.5

Numbers (*n*) are unweighted counts, and percentages (%) are weighted to represent the national population with 95% confidence intervals (CI).

Table 3 presents the bivariate associations between background characteristics and patterns of tobacco use. All background characteristics showed statistically significant associations with patterns of tobacco use (*p* < 0.05), with particularly strong associations observed for age, sex, education, marital status, occupation, wealth quintile, and exposure to mass media (*p* < 0.001). No cases of smokeless or dual tobacco use were observed among respondents with higher education in the descriptive analysis.

**Table 3:** Bivariate (chi-square) associations between background characteristics and patterns of tobacco use.

Variable	Tobacco Use				*p* value
	No tobacco use	Smoked tobacco use	Smokeless tobacco use	Dual tobacco use	
	*N* (%)	*N* (%)	*N* (%)	*N* (%)	
Age					<0.001^**^
15–19	2,402 (93.0)	196 (6.8)	3 (0.1)	2 (0.1)	
20–29	4,467 (87.9)	657 (11.6)	12 (0.2)	13 (0.3)	
30–39	4,437 (85.6)	754 (13.6)	25 (0.5)	17 (0.3)	
40–49	3,727 (81.7)	865 (17.4)	24 (0.5)	19 (0.4)	
Sex					<0.001^**^
Female	12,312 (96.3)	547 (3.4)	21 (0.2)	3 (0.1)	
Male	2,721 (59.1)	1,925 (38.9)	43 (0.8)	48 (1.2)	
Education					<0.001^**^
No education	1,651 (78.9)	480 (20.2)	12 (0.5)	8 (0.4)	
Primary	5,718 (85.9)	983 (13.2)	35 (0.5)	24 (0.4)	
Secondary	6,119 (87.4)	905 (12.1)	17 (0.2)	19 (0.3)	
Higher	1,543 (93.5)	105 (6.5)	0 (0.0)	0 (0.0)	
Residence					0.001^*^
Rural	10,539 (85.6)	1,871 (13.5)	57 (0.5)	47 (0.4)	
Urban	4,494 (88.1)	601 (11.6)	7 (0.2)	4 (0.1)	
Marital status					<0.001^**^
Never married	5,208 (90.4)	609 (9.3)	16 (0.2)	8 (0.1)	
Married	8,982 (84.2)	1,717 (15.0)	47 (0.4)	38 (0.4)	
Not currently married or cohabiting^a^	843 (85.4)	146 (13.8)	1 (0.1)	5 (0.7)	
Occupation					<0.001^**^
Not working	3,764 (94.8)	232 (5.0)	5 (0.1)	1 (0.1)	
Agriculture/self-employed	2,456 (81.0)	623 (16.9)	22 (0.8)	33 (1.3)	
Clerical/sales/services	2,797 (91.5)	229 (8.2)	9 (0.3)	0 (0.0)	
Professional/technical/ managerial	1,033 (86.9)	146 (12.9)	1 (0.1)	1 (0.1)	
Skilled manual	1,388 (78.7)	446 (20.9)	9 (0.2)	5 (0.2)	
Unskilled manual	3,564 (82.4)	788 (16.9)	18 (0.4)	11 (0.3)	
Wealth quintile					<0.001^**^
Lowest	2,548 (79.5)	687 (19.6)	20 (0.5)	13 (0.4)	
Second	2,813 (84.7)	532 (14.1)	25 (0.7)	14 (0.5)	
Middle	3,146 (87.3)	479 (12.0)	8 (0.2)	14 (0.5)	
Fourth	3,279 (88.6)	441 (11.0)	8 (0.3)	6 (0.1)	
Highest	3,247 (90.2)	333 (9.6)	3 (0.1)	4 (0.1)	
Exposure to mass media					<0.001^**^
No exposure	1,768 (84.9)	345 (14.8)	7 (0.2)	3 (0.1)	
Low exposure	3,018 (83.9)	602 (15.4)	19 (0.4)	11 (0.3)	
High exposure	10,247 (87.2)	1,525 (12.0)	38 (0.3)	37 (0.3)	

Numbers (N) are unweighted counts, and percentages (%) are weighted to represent the national population with 95% confidence intervals (CI). *P* values are obtained from chi-squared test accounting for survey design. ^*^Statistically significant: *p* < 0.05. ^**^Highly statistically significant: *p* < 0.001. Cells with fewer than 25 unweighted cases may yield unstable weighted estimates and should be interpreted with caution, consistent with GTSS/GATS reporting recommendations. ^*a*^Includes widowed, divorced, and separated/no longer living together.

Table 4 presents the results of the survey-weighted multivariable multinomial logistic regression analysis of factors associated with patterns of tobacco use, using no tobacco use specified as the reference category. Older age was associated with higher likelihood of smoked tobacco use, with respondents aged 40–49 years being more likely to use smoked tobacco compared with those aged 15–19 years (ARRR = 3.58, 95% CI 2.70, 4.74; *p* < 0.001). Male respondents were substantially more likely than females to use smoked tobacco (ARRR = 21.96, 95% CI 18.38, 26.22; *p* < 0.001), smokeless tobacco (ARRR = 9.62, 95% CI 4.76, 19.45; *p* < 0.001), and dual tobacco use (ARRR = 55.01, 95% CI 14.95, 202.43; *p* < 0.001). The ARRR estimate for dual tobacco use among men should be interpreted cautiously because of the wide confidence interval and small number of dual users. Higher educational attainment and higher household wealth were consistently associated with a lower likelihood of smoked tobacco use. Because no smokeless or dual tobacco use was observed among respondents with higher education (Table 3), the corresponding estimates were near zero due to complete separation and are not interpretable as effect sizes. After adjustment for sociodemographic factors, urban residence was associated with a higher likelihood of smoked tobacco use compared with rural residence (ARRR = 1.28, 95% CI 1.06, 1.54; *p* = 0.010). Being not currently married or cohabiting, which includes widowed, divorced, and separated individuals, was associated with a higher likelihood of smoked tobacco use (ARRR = 1.82, 95% CI 1.34, 2.46; *p* < 0.001) and dual tobacco use (ARRR = 9.68, 95% CI 2.21, 42.34; *p* = 0.003). Other sociodemographic variables, including occupation and exposure to mass media, did not show statistically significant associations with most tobacco use patterns after adjustment.

**Table 4:** Survey-weighted multivariable multinomial logistic regression analysis of factors associated with patterns of tobacco use.

Variable	Tobacco use					
	Smoked tobacco use vs. no tobacco use		Smokeless tobacco use vs. no tobacco use		Dual tobacco use vs. no tobacco use	
	ARRR (95% CI)	*p* value	ARRR (95% CI)	*p* value	ARRR (95% CI)	*p* value
Age		<0.001^**^		0.059		0.314
15–19	1 (ref:)		1 (ref:)		1 (ref:)	
20–29	2.17 (1.71, 2.74)	< 0.001^**^	3.23 (0.58, 17.89)	0.178	2.31 (0.39, 13.61)	0.354
30–39	2.62 (1.98, 3.46)	< 0.001^**^	7.69 (1.36, 43.39)	0.021^*^	2.31 (0.37, 14.52)	0.369
40–49	3.58 (2.70, 4.74)	< 0.001^**^	9.94 (1.45, 68.15)	0.019^*^	3.06 (0.56, 16.75)	0.197
Sex		<0.001^**^		<0.001^**^		<0.001^**^
Female	1 (ref:)		1 (ref:)		1 (ref:)	
Male	21.96 (18.38, 26.22)	< 0.001^**^	9.62 (4.76, 19.45)	< 0.001^**^	55.01 (14.95, 202.43)	< 0.001^**^
Education		<0.001^**^		<0.001^**^		<0.001^**^
No education	1 (ref:)		1 (ref:)		1 (ref:)	
Primary	0.61 (0.49, 0.77)	< 0.001^**^	0.83 (0.34, 2.02)	0.679	0.71 (0.28, 1.81)	0.470
Secondary	0.55 (0.43, 0.72)	< 0.001^**^	0.60 (0.23, 1.61)	0.312	0.64 (0.26, 1.56)	0.325
Higher	0.38 (0.27, 0.54)	< 0.001^**^	1.25 × 10^−7^ (3.68 × 10^−8^, 4.23 × 10^−7^)	< 0.001^**^	5.45 × 10^−7^ (1.63 × 10^−7^, 1.83 × 10^−6^)	< 0.001^**^
Residence		0.010^*^		0.976		0.514
Rural	1 (ref:)		1 (ref:)		1 (ref:)	
Urban	1.28 (1.06, 1.54)	0.010^*^	0.99 (0.37, 2.59)	0.976	0.52 (0.07, 3.77)	0.514
Marital status		<0.001^**^		0.637		0.005^*^
Never married	1 (ref:)		1 (ref:)		1 (ref:)	
Married	1.12 (0.94, 1.33)	0.208	0.74 (0.31, 1.77)	0.499	2.24 (0.82, 6.14)	0.116
Not currently married or cohabiting^a^	1.82 (1.34, 2.46)	< 0.001^**^	0.36 (0.04, 3.23)	0.358	9.68 (2.21, 42.34)	0.003^*^
Occupation		0.212		0.370		<0.001^**^
Not working	1 (ref:)		1 (ref:)		1 (ref:)	
Agriculture/self-employed	0.81 (0.63, 1.03)	0.079	1.71 (0.47, 6.16)	0.410	9.07 (1.24, 66.39)	0.030^*^
Clerical/sales/services	0.96 (0.74, 1.24)	0.738	2.01 (0.45, 8.84)	0.359	1.18 × 10^−6^ (1.52 × 10^−7^, 9.14 × 10^−6^)	< 0.001^**^
Professional/technical/ managerial	0.99 (0.72, 1.37)	0.968	0.28 (0.03, 2.91)	0.288	2.17 (0.13, 35.44)	0.585
Skilled manual	1.06 (0.83, 1.36)	0.625	0.68 (0.15, 3.10)	0.618	1.44 (0.15, 14.00)	0.754
Unskilled manual	1.01 (0.80, 1.28)	0.931	1.11 (0.30, 4.12)	0.875	2.86 (0.34, 23.89)	0.332
Wealth quintile		<0.001^**^		0.003^*^		0.537
Lowest	1 (ref:)		1 (ref:)		1 (ref:)	
Second	0.65 (0.52, 0.81)	< 0.001^**^	0.99 (0.48, 2.04)	0.971	0.83 (0.30, 2.31)	0.720
Middle	0.53 (0.42, 0.67)	< 0.001^**^	0.34 (0.14, 0.82)	0.017^*^	0.93 (0.42, 2.04)	0.852
Fourth	0.46 (0.37, 0.58)	< 0.001^**^	0.38 (0.16, 0.92)	0.032^*^	0.46 (0.18, 1.21)	0.115
Highest	0.38 (0.30, 0.50)	< 0.001^**^	0.16 (0.06, 0.46)	0.001^*^	0.71 (0.13, 3.86)	0.693
Exposure to mass media		0.223		0.133		0.058
No exposure	1 (ref:)		1 (ref:)		1 (ref:)	
Low exposure	0.92 (0.74, 1.14)	0.453	1.72 (0.56, 5.26)	0.344	3.25 (0.48, 21.73)	0.224
High exposure	0.88 (0.71, 1.08)	0.223	2.15 (0.79, 5.83)	0.133	4.77 (0.95, 23.89)	0.058

^*a*^Includes widowed, divorced, and separated/no longer living together. ^*^Statistically significant: *p* < 0.05; ^**^highly statistically significant: *p* < 0.001; ARRR, adjusted relative risk ratio; CI, Confidence interval; Reference outcome category: “no tobacco use”. The multivariable multinomial logistic regression analysis was adjusted for age, sex, education, residence, marital status, occupation, wealth quintile, and exposure to mass media. All analyses accounted for survey weights. ARRR estimates presented in scientific notation reflect statistical instability due to complete or near-complete separation and sparse subgroup counts and should not be interpreted substantively.

## 4 Discussion

Using nationally representative data from the Myanmar Demographic and Health Survey (MDHS) 2015–2016 (8), this study provides baseline, product-specific evidence on tobacco use in Myanmar prior to recent policy and market developments. By distinguishing smoked, smokeless, and dual tobacco use, the analysis provides nationally representative baseline estimates of product-specific tobacco use patterns during the survey period. As the MDHS 2015–2016 remains the most recent nationally representative dataset with adult tobacco use indicators in Myanmar, these findings provide an important reference point for monitoring trends and informing tobacco control strategies. However, the findings should be interpreted within the context of the survey period, as tobacco use patterns and product availability may have changed over time due to evolving tobacco control policies, market dynamics, and social factors.

Most respondents (86.3%) reported no current tobacco use, while 13.0% used smoked tobacco and only small proportions used smokeless tobacco limited to chewing tobacco and snuff (0.4%) or dual tobacco (0.3%). The very low prevalence of smokeless and dual tobacco use should be interpreted cautiously due to the small number of cases and potential measurement limitations in the MDHS, particularly the incomplete capture of betel quid with tobacco. This observed pattern may reflect the widespread availability of low-cost combustible tobacco products in Myanmar, particularly locally produced cheroots. Higher smoking prevalence (26.1%) and smokeless use (43.2%) reported in the 2014 Myanmar WHO STEPS survey (12) likely reflect differences in age coverage, as STEPs includes older adults, as well as methodological variations between surveys, consistent with the age gradient observed in our analysis. The 2016 Myanmar Global Youth Tobacco Survey (9) likewise showed higher cigarette smoking than smokeless use among adolescents, reinforcing the dominance of smoked products. Nationally representative findings from Cambodia (11) similarly indicate that smoked tobacco is the dominant form of use. These findings indicate that combustible tobacco products accounted for most tobacco use in the study population during the survey period, highlighting the need for product-specific surveillance and continued focus on combustible tobacco in national tobacco control efforts.

Older age was strongly associated with smoked tobacco use, with individuals aged 40–49 years having more than three times the likelihood of smoking compared with those aged 15–19 years (ARRR = 3.58, 95% CI: 2.70, 4.74, *p* < 0.001). This observed age gradient is consistent with nationally representative findings from Cambodia (11), India (13), and mainland China (14), which demonstrate higher smoking prevalence among older adults. The observed gradient may be related to cumulative uptake and sustained smoking across adulthood, differential cessation patterns, or generational (cohort) differences in tobacco exposure. However, the cross-sectional design precludes disentangling age, period, and cohort effects. Although adolescence remains a critical window for prevention, the higher prevalence of smoked tobacco use among older adults highlights the importance of accessible cessation support across the adult life course. Longitudinal research is needed to clarify smoking trajectories and cessation dynamics in Myanmar.

Male respondents were markedly more likely than females to use smoked tobacco (ARRR = 21.96, 95% CI: 18.38, 26.22, *p* < 0.001), as well as smokeless (ARRR = 9.62, 95% CI: 4.76, 19.45, *p* < 0.001) and dual tobacco (ARRR = 55.01, 95% CI: 14.95, 202.43, *p* < 0.001), highlighting the highly gendered nature of tobacco use in Myanmar. The very large relative risk estimate for dual use, accompanied by a wide confidence interval, should be interpreted cautiously given the small number of dual users, which may also contribute to limited precision, sparse-data instability, and residual confounding despite the use of survey-weighted models. This considerable gender disparity in smoked tobacco use aligns with nationally representative findings from mainland China (14) and Cambodia (11) and with other regional studies, including multi-city Chinese analyses (15) and Thai university samples (16), consistently demonstrating substantially higher smoking prevalence among men. Such patterns may partly reflect gender differences in the social acceptability of smoking and tobacco-related norms within male social and occupational environments in Southeast Asia (17). These findings suggest the potential importance of gender-responsive tobacco control strategies in Myanmar. From a programmatic perspective, this may include prioritizing male-focused prevention and cessation interventions, particularly in male-dominated workplaces and community settings where smoking may be socially reinforced, while sustaining low smoking prevalence among women. Ongoing surveillance among women is also essential to detect shifts in smoking patterns (17).

Higher educational attainment was inversely associated with smoked tobacco use; individuals with higher education had a 62% lower likelihood of smoking compared with those with no education (ARRR = 0.38, 95% CI 0.27, 0.54, *p* < 0.001). This inverse pattern aligns with nationally representative findings from Cambodia (11), a comparable lower-middle-income country, and from high-income settings such as the United States (18), where smoking initiation and prevalence are consistently lower among individuals with higher educational attainment. This gradient may reflect differences in health literacy, access to information, and responsiveness to tobacco control messaging. Evidence from Myanmar school settings documenting suboptimal awareness of tobacco control regulations and penalties among adolescents (19) further highlights the importance of accessible and clearly communicated tobacco control policies. These findings suggest the potential value of strengthening education-sensitive tobacco control strategies in Myanmar, including simplified health communication and enhanced integration of cessation support within primary healthcare and community-based platforms to better reach individuals with lower educational attainment.

Smoking exhibited a clear inverse wealth gradient, with progressively lower likelihood among more affluent groups. This pattern aligns with Cambodia national findings (11) and is consistent with persistent economic inequalities in tobacco use. The concentration of smoking among lower-wealth populations may reflect structural disparities in exposure, access to information, and cessation resources. These findings suggest the potential importance of incorporating an equity perspective within tobacco control strategies in Myanmar, including targeted communication and strengthened cessation support integrated into primary healthcare and community-based services. After adjustment for sociodemographic factors, urban residence was associated with a higher likelihood of smoked tobacco use, although crude prevalence was higher in rural areas. Despite the inverse wealth gradient observed in our study, Cambodia (11) reported no urban-rural difference in smoking, suggesting that urban-rural patterns may vary across settings. Individuals who were not currently married or cohabiting had higher likelihoods of smoked tobacco use and dual use compared with those never married. In contrast, marital status was not significantly associated with tobacco use in Cambodia (11), suggesting contextual variation in this relationship. Although the cross-sectional design limits causal inference, the observed association may be related to differences in social support, stress exposure, or household context across marital groups. The elevated dual use in this subgroup should be interpreted cautiously given small case numbers but merits further investigation. Future studies with more stable subgroup distributions may benefit from interaction or stratified analyses to further explore sociodemographic disparities in tobacco use.

### 4.1 Strengths of the study

Our study has important strengths. It is based on nationally representative MDHS data with rigorous two-stage stratified sampling and standardized field procedures, enhancing generalizability to the Myanmar population aged 15–49 years. The application of survey weights and adjustment for clustering and stratification ensured valid population-level inference. Importantly, this study adopted a product-specific classification framework and employed multinomial logistic regression to examine smoked, smokeless, and dual tobacco use as distinct outcomes, thereby preserving heterogeneity in tobacco use patterns rather than aggregating them into a single binary measure. Although the analysis used an existing nationally representative dataset, the present study specifically focused on product-specific tobacco classification and population-level distribution of smoked, smokeless, and dual tobacco use. This approach extends prior Myanmar DHS analyses that combined tobacco with other substances such as betel quid or primarily reported sex-specific prevalence estimates. In addition, careful assessment of multicollinearity and examination of unweighted cell counts enhanced analytical transparency and robustness. Together, these methodological features provide a comprehensive and policy-relevant assessment of tobacco use patterns and their sociodemographic distribution in Myanmar during the survey period.

### 4.2 Limitations of the study

This study has several limitations. First, the cross-sectional design of the MDHS cannot establish causal inference and limits the ability to disentangle age, period, and cohort effects underlying observed associations. Therefore, the observed associations should be interpreted as correlational rather than causal. Second, the analysis is based on data from 2015–2016, and more recent nationally representative data on adult tobacco use in Myanmar remain limited; therefore, the findings may not fully reflect current tobacco use patterns, product availability, or recent policy and social changes. Third, the MDHS age restriction to 15–49 years excludes adults aged ≥ 50 years, a subgroup with potentially different and higher tobacco use prevalence and cumulative exposure, which may underestimate the overall population burden. As tobacco use is age-dependent, exclusion of older adults limits generalizability to the broader adult population of Myanmar, and the findings should therefore be interpreted only for individuals aged 15–49 years. Fourth, tobacco use was based on self-report and may therefore be subject to information and potential misclassification bias, including recall bias and social desirability bias. The absence of biochemical validation may further limit the accuracy of exposure measurement. Underreporting may be more common among women due to sociocultural norms discouraging female tobacco use in Myanmar, potentially resulting in underestimated prevalence. Fifth, the very low prevalence of smokeless and dual tobacco use resulted in small cell counts in some subgroups, producing wide confidence intervals, unstable relative risk estimates, and limited precision despite appropriate application of survey weights. Accordingly, estimates for these categories may be sensitive to sparse-data bias and should be interpreted cautiously with respect to model stability and effect size magnitude. The absence of smokeless and dual tobacco users in the higher-education subgroup also resulted in near-zero ARRR estimates due to complete separation; these findings should therefore be interpreted cautiously. Sixth, the MDHS does not capture detailed measures of frequency, intensity, duration of use, or age at initiation, nor does it include emerging products such as electronic cigarettes, limiting assessment of cumulative exposure and evolving product transitions. In addition, the product-specific categories did not capture differences in nicotine exposure across tobacco products, which may limit interpretation of comparative health risks. Seventh, although a variable for betel quid with tobacco exists in the MDHS, it contained no usable data in the analyzed dataset and was not available for men; therefore, smokeless tobacco in our study was limited to chewing tobacco and snuff, which may underestimate the true prevalence of smokeless tobacco use in Myanmar. Finally, although multiple sociodemographic characteristics were adjusted for, residual confounding by unmeasured behavioral, cultural, or contextual factors cannot be excluded. In addition, interaction effects and stratified relationships between covariates were not examined and may warrant further investigation.

## 5 Conclusions

Our study, using nationally representative data from individuals aged 15–49 years in the 2015–2016 Myanmar Demographic and Health Survey, found that smoked tobacco predominated among individuals aged 15–49 years in Myanmar, while smokeless and dual use were comparatively uncommon. Marked sociodemographic inequalities were observed across sex, age, education, and wealth groups. By distinguishing smoked, smokeless, and dual tobacco use, this study suggests marked sociodemographic gradients in tobacco exposure during the 2015–2016 survey period and highlights the need for updated, product-specific surveillance in Myanmar. These findings provide nationally representative baseline evidence on tobacco use patterns in Myanmar during the survey period and support the importance of improved and updated surveillance to monitor changes over time.

## Data Availability

The datasets analyzed in this study are not readily available from the authors because the 2015–2016 Myanmar Demographic and Health Survey (MDHS) data are available from the Demographic and Health Surveys (DHS) Program upon registration and approval. Requests to access the datasets should be directed to the DHS Program Data Access portal: https://www.dhsprogram.com/data/available-datasets.cfm.
