# Supplementary material for: Sociodemographic inequalities in product-specific tobacco use in Myanmar: a nationally representative study of the 2015–2016 demographic and health survey
Source: Front Public Health. 2026 Jun 23;14:1839321. doi: 10.3389/fpubh.2026.1839321 (PMC13337633; doi:10.3389/fpubh.2026.1839321)
Supplement: Supplementary file 1 [file Table_1.docx]

**S1 Table:** Bivariable Multinomial Logistic Regression of Factors Associated with Tobacco Use

| **Variable** | **Tobacco Consumption** | | | | | |  |
| --- | --- | --- | --- | --- | --- | --- | --- |
|  | **Smoked tobacco use versus No tobacco use** | | **Smokeless tobacco use versus No tobacco use** | | **Dual tobacco use versus No tobacco use** | |  |
|  |  |  |  |  |  |  |  |
|  | **RRR (95% CI)** | ***p* value** | **RRR (95% CI)** | ***p* value** | **RRR  (95% CI)** | ***p* value** |  |
| **Age** |  | < 0.001** |  | 0.091 |  | 0.028* |  |
| 15-19 | 1 (ref:) |  | 1 (ref:) |  | 1 (ref:) |  |  |
| 20-29 | 1.81 (1.49, 2.18) | < 0.001** | 2.30 (0.43, 12.15) | 0.328 | 3.18 (0.69, 14.60) | 0.136 |  |
| 30-39 | 2.16 (1.77, 2.65) | <0.001** | 5.32 (1.03, 27.35) | 0.045* | 3.97 (0.86, 18.30) | 0.077 |  |
| 40-49 | 2.91 (2.39, 3.53) | <0.001** | 6.22 (1.21, 31.92) | 0.029* | 5.36 (1.40, 20.50) | 0.014* |  |
| **Sex** |  | < 0.001** |  | < 0.001** |  | < 0.001** |  |
| Female | 1 (ref:) |  | 1 (ref:) |  | 1 (ref:) |  |  |
| Male | 18.07 (15.12, 21.59) |  | 8.52 (4.47, 16.23) |  | 65.92 (18.50, 234.88) |  |  |
| **Education** |  | < 0.001** |  | < 0.001** |  | < 0.001** |  |
| No education | 1 (ref:) |  | 1 (ref:) |  | 1 (ref:) |  |  |
| Primary | 0.60 (0.51, 0.72) | < 0.001** | 0.98 (0.43, 2.25) | 0.958 | 0.87 (0.33, 2.32) | 0.778 |  |
| Secondary | 0.54 (0.45, 0.65) | < 0.001** | 0.48 (0.18, 1.31) | 0.153 | 0.62 (0.23, 1.68) | 0.351 |  |
| Higher | 0.27 (0.21, 0.36) | < 0.001** | 4.79×10^-8^ (2.33×10^-8^, 9.88×10^-8^) | < 0.001** | 4.88×10^-8^ (2.14×10^-8^, 1.11×10^-7^) | < 0.001** |  |
| **Residence** |  | 0.010* |  | 0.099 |  | 0.012* |  |
| Rural | 1 (ref:) |  | 1 (ref:) |  | 1 (ref:) |  |  |
| Urban | 0.84 (0.73, 0.96) |  | 0.35 (0.09, 1.22) |  | 0.13 (0.03, 0.64) |  |  |
| **Marital status** |  | < 0.001** |  | 0.077 |  | 0.005* |  |
| Never married | 1 (ref:) |  | 1 (ref:) |  | 1 (ref:) |  |  |
| Married | 1.73 (1.54, 1.95) | < 0.001** | 1.96 (1.02, 3.79) | 0.045* | 3.28 (1.47, 7.33) | 0.004* |  |
| Not currently married or cohabiting^a^ | 1.57 (1.21, 2.03) | 0.001* | 0.65 (0.08, 5.12) | 0.686 | 5.27 (1.81, 15.36) | 0.002* |  |
| **Occupation** |  | < 0.001** |  | < 0.001** |  | < 0.001** |  |
| Not working | 1 (ref:) |  | 1 (ref:) |  | 1 (ref:) |  |  |
| Agriculture/ Self-employed | 3.90 (3.15, 4.82) | < 0.001** | 7.92 (2.70, 23.24) | < 0.001** | 94.89 (12.65, 711.66) | < 0.001** |  |
| Clerical/ sales/ services | 1.68 (1.31, 2.16) | < 0.001** | 2.60 (0.58, 11.56) | 0.209 | 1.43×10^-6^ (2.00×10^-7^, 1.02×10^-5^) | < 0.001** |  |
| Professional/ technical/   managerial | 2.78 (1.99, 3.86) | < 0.001** | 0.35 (0.04, 3.33) | 0.361 | 8.77 (0.54, 142.97) | 0.127 |  |
| Skilled manual | 4.97 (3.95, 6.25) | < 0.001** | 2.10 (0.56, 7.86) | 0.272 | 12.84 (1.33, 124.25) | 0.028* |  |
| Unskilled manual | 3.84 (3.10, 4.75) | < 0.001** | 3.90 (1.36, 11.20) | 0.011* | 18.78 (2.32, 152.23) | 0.006* |  |
| **Wealth quintile** |  | < 0.001** |  | 0.013* |  | 0.040* |  |
| Lowest | 1 (ref:) |  | 1 (ref:) |  | 1 (ref:) |  |  |
| Second | 0.68 (0.58, 0.79) | < 0.001** | 1.19 (0.56, 2.49) | 0.652 | 1.06 (0.39, 2.86) | 0.902 |  |
| Middle | 0.56 (0.47, 0.67) | < 0.001** | 0.41 (0.17, 0.97) | 0.044* | 1.10 (0.48, 2.54) | 0.821 |  |
| Fourth | 0.50 (0.42, 0.59) | < 0.001** | 0.42 (0.17, 1.05) | 0.063 | 0.40 (0.16, 1.05) | 0.063 |  |
| Highest | 0.43 (0.36, 0.52) | < 0.001** | 0.13 (0.03, 0.64) | 0.012* | 0.26 (0.06, 1.21) | 0.085 |  |
| **Exposure to mass media** |  | 0.010* |  | 0.421 |  | 0.090 |  |
| No exposure | 1 (ref:) |  | 1 (ref:) |  | 1 (ref:) |  |  |
| Low exposure | 1.05 (0.87, 1.28) | 0.600 | 1.85 (0.68, 5.03) | 0.227 | 4.58 (0.68, 30.90) | 0.118 |  |
| High exposure | 0.79 (0.67, 0.95) | 0.010* | 1.45 (0.59, 3.58) | 0.421 | 4.49 (0.79, 25.57) | 0.090 |  |

**Highly significance: *p* < 0.001; *Significance: *p* < 0.05; RRR: Unadjusted relative risk ratio; All analyses used survey weights; CI: Confidence interval; Ref: “no tobacco use” as the reference category
